# Prevalence of malaria relapse: systematic review with meta-analysis

**DOI:** 10.1590/1518-8345.2619.3111

**Published:** 2019-03-18

**Authors:** Talita Lima do Nascimento, Suleima Pedroza Vasconcelos, Yara Peres, Mirla Jéssica Sampaio de Oliveira, Monica Taminato, Káren Mendes Jorge de Souza

**Affiliations:** 1 Universidade Federal do Acre, Centro de Ciências da Saúde e do Desporto, Rio Branco, AC, Brazil.; 2 Universidade Federal de São Paulo, Escola Paulista de Enfermagem, São Paulo, SP, Brazil.; 3 Secretaria Municipal de Saúde de Pauiní, Unidade Básica Saúde José Roberto, Pauiní, AM, Brazil.; 4 Secretaria Municipal de Saúde de Cruzeiro do Sul, Unidade de Saúde da Família 25 de agosto, Cruzeiro do Sul, AC, Brazil.

**Keywords:** Malaria, Recurrence, Prevalence, Neglected Diseases, Public Health Nursing, Malária, Recaída, Prevalência, Doenças Negligenciadas, Enfermagem em Saúde Pública, Malaria, Recurrencia, Prevelencia, Enfermedades Desatendidas, Enfermería en Salud Pública

## Abstract

**Objective:**

systematic review with a meta-analysis of the prevalence of malaria relapse.

**Method:**

it consisted in a search for cross-sectional studies, carried out in three databases, without application of filters. A total of 1,924 articles were identified, selected based on eligibility criteria. The selection was made in pairs from the reading of the titles, abstracts and text. The meta-analysis was performed with a statistical program.

**Results:**

a sample of 1,308 patients with malaria, ranging from 70 to 586 patients in the study. Relapse was estimated at 0.47%, with a 95% confidence interval and 99.04% of squared *i*. In the included studies, the prevalence of relapse ranged from 17.00% to 92.85%. The result of the meta-analysis is considered relevant, despite the heterogeneity.

**Conclusion:**

relapse is a phenomenon that can contribute to the maintenance of the endemicity of malaria in the world and to introduce it in non-affected areas. In addition, there is the need for advancement in the production of knowledge regarding this disease, to qualify the research methods on prevalence.

## RESUMO

**Objetivo:**

realizar uma revisão sistemática com metanálise da prevalência de recaída por malária.

**Método:**

consistiu na busca por estudos transversais, realizada em três bases de dados, sem aplicação de filtros. Foram identificados 1.924 artigos, selecionados a partir de critérios de elegibilidade. A seleção foi realizada em par na sequência de leitura dos títulos, resumos e texto. A metanálise foi realizada com programa estatístico.

**Resultados:**

uma amostra de 1.308 pacientes com malária, variando de 70 a 586 pacientes nos estudo. A recaída foi estimada em 0,47%, com intervalo de confiança de 95% e i quadrado de 99,04%. Nos estudos incluídos, a prevalência de recaída variou de 17,00% a 92,85%. Considera-se o resultado da metanálise relevante, apesar da heterogeneidade.

**Conclusão:**

a recaída é um fenômeno que pode contribuir para a manutenção da endemicidade da malária no mundo, além de poder introduzi-la em áreas não afetadas. Além disso, há necessidade, para avanço na produção de conhecimento referente a essa doença, de qualificar os métodos de pesquisa sobre prevalência.

## RESUMEN

**Objetivo:**

realizar una revisión sistemática con metaanálisis de la prevalencia de recaída por malaria.

**Método:**

consistió en la búsqueda por estudios transversales, realizada en tres bases de datos, sin aplicación de filtros. Fueron identificados 1.924 artículos, seleccionados a partir de criterios de elegibilidad. La selección fue realizada en pares en la secuencia de lectura de los títulos, resúmenes y texto. El metaanálisis fue realizado con un programa estadístico.

**Resultados:**

una muestra de 1.308 pacientes con malaria, variando de 70 a 586 pacientes en el estudio. La recaída fue estimada en 0,47%, con intervalo de confianza de 95% e i cuadrado de 99,04%. En los estudios incluidos, la prevalencia de recaída varió de 17,00% a 92,85%. Se considera el resultado de la metaanálisis relevante, a pesar de la heterogeneidad.

**Conclusión:**

la recaída es un fenómeno que puede contribuir para el mantenimiento de la endemicidad de la malaria en el mundo, además de poder introducirla en áreas no afectadas. Además de eso, es necesario de calificar los métodos de investigación sobre prevalencia para el avance en la producción de conocimiento referente a esa enfermedad.

## Introduction

Malaria is recognized by the World Health Organization (WHO) as an important public health problem because of its high prevalence and because it is related to low socioeconomic development^1^. The tropical and subtropical areas of the planet are the most affected, most notably Southeast Asia, the Amazon and Africa. This latter has 80% of the total cases and deaths by malaria in the world^2^.

The disease affects, especially, poor populations with difficult access to health services, in poor housing and work conditions. High incidence is also observed in areas of disorderly occupation of territories and in the migration from the rural to the periphery of cities^3^.

Malaria is considered a neglected tropical disease whose management and clinical management need to be more effective in the field of public health^4^. Add to this the problem of disease relapse, which has contributed to its endemicity. Thus, it is worth reflecting which challenges and coping strategies are most feasible for malaria control, as well as the role of nursing in identifying and developing alternatives to deal with disease relapse^4^

Understanding the biological cycle of malaria is important in understanding the phenomenon of relapse. The disease is caused by protozoa of the genus *Plasmodium* of four species, and the most prevalent infection is by *Plasmodium vivax*, which is also the type responsible for most cases of relapse, since it develops latent forms in the liver cells called *hypnozoites*, which may stay inactive for weeks^5^ ^-^ ^6^. 

Malaria relapse can be defined as the recurrence of asexual parasitemia after drug treatment and the finding of its negativity in a given period of time, due to a variety of factors^7^.

According to the scientific literature, this clinical picture can occur for several reasons, among which the following stand out: lack of patient adherence to treatment, prescription of therapeutic regimens that do not combat the tissue phase of the cycle, resistance of *Plasmodium* to antimalarial drugs and reactivation of the *hypnozoites* ^8^.

The phenomenon of relapse has important consequences for the patient, the community and the health services, as it contributes to the maintenance of the number of cases reported, thus increasing public health costs and expenses^9^. 

Investigating and measuring the prevalence rate of malaria relapse is an important indicator for health services and for disease control programs. This information can help nurses in the planning of interventions, guide managers in the application of financial resources, and foster research needed to explain the factors that determine the phenomenon at the local level and its endemic permanence^5^ ^,^ ^10^. In addition, this may be an important data for conducting economic evaluations studies in malaria control programs. 

The objective of this study was to perform a systematic review, with meta-analysis, of observational studies that estimated the prevalence of malaria relapse. 

## Method

This is a systematic review with a meta-analysis of observational studies, conducted according to the recommendations of the Preferred Reporting Items for Systematic Reviews and Meta-Analyzes (PRISMA)^11^. The study was guided by a structured question in the format of structured clinical question, as follows: population of interest or health problem (P), which corresponds to patients with malaria; intervention (I): malaria control programs; comparator (C): not applicable; outcome (O): prevalence of relapse; and study (S): observational studies.

The search and selection of scientific evidence for the review was carried out in the databases Medical Literature Analysis and Retrieval System Online (Medline) via National Library of Medicine (Pubmed), Scientific and Technical Literature of Latin America and the Caribbean (Lilacs) and Cochrane Library. The bases were chosen based on the wide coverage of the Pubmed and Cochrane Library and on the location of studies carried out in the Americas, endemic for malaria, in the case of Lilacs.

**Figure 1:**
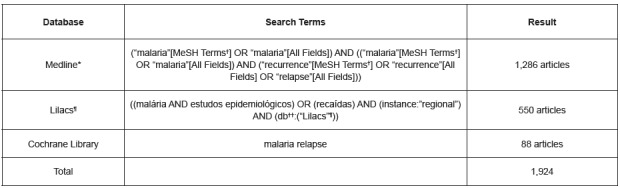
Search strategy by database

Figure 1 describes the search strategy used in each database. 

The search in the databases was carried out in August 2016. No filters were applied so that we could identify all the articles available in the databases and reduce the risk of publication bias. The eligibility criteria were being cross-sectional observational studies and having outcome relapse prevalence as outcome. 

The reading and selection of the articles was carried out by two reviewers independently, starting by the titles, followed by the abstracts and full texts. In the selection of the titles, we included all those that presented one of the following terms: relapse, prevalence, malaria and *Plasmodium*. When the application of the eligibility criteria was not sufficient to establish whether or not the article would be included in a phase, it was retained for reading the abstract. The last phase of the selection was made by reading the full text of the articles. 

*Medline - Medical Literature Analysis and Retrieval System Online; †MeSH Terms - Terms of Medical Subject Headings; Lilacs - Latin America and Caribbean Literature in Health Sciences; ††db - database

In the seven selected studies, an analysis was carried out to verify the methodological quality, using the checklist STRENBE (Strengthening the Reporting of Observational Studies in Epidemiology), as they were cross-sectional studies^12^. Twenty-two items are referred to in this instrument, which should be reported, improving methodological rigor in this type of design. At this stage, two studies were excluded for not presenting key elements to the study design and because the source for obtaining the data to estimate the prevalence rate was secondary. In the five eligible studies, when analyzing the checklist, weaknesses were detected in the description of the measures to reduce risk of bias. 

Data extraction from the included studies was performed in pairs independently, using a previously planned spreadsheet, containing the following information: author, year of publication, period of data collection, study location, age group, method used for case detection, sample (n) and prevalence of outcome. 

**Figure 2:**
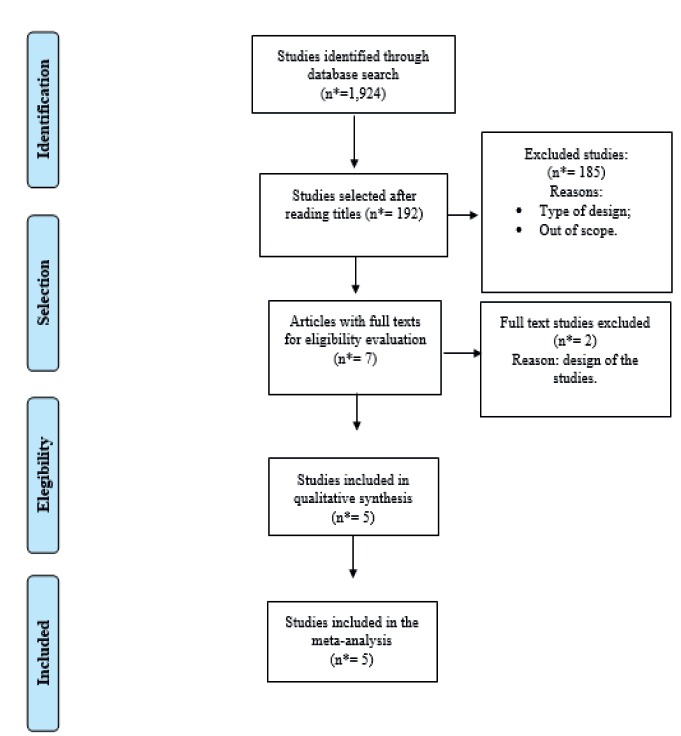
Study selection flowchart

*n - Sample

**Figure 3:**
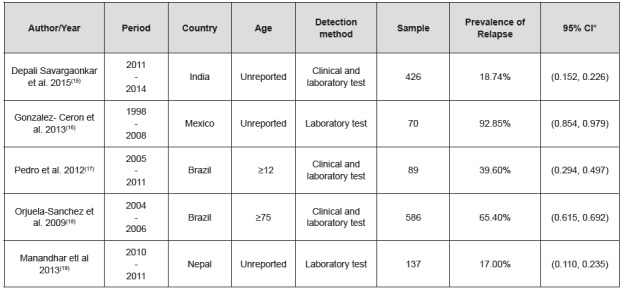
Data extracted from the studies included in the systematic review

*CI - Confidence Interval

**Figure 4:**
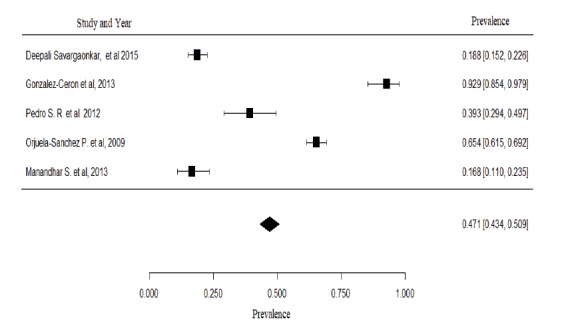
Meta-analysis of the prevalence of relapse

Meta-analysis is a statistical analysis that combines and synthesizes results from several studies, and makes it possible to explain the differences in their findings. The measure of summarization of the results for the meta-analysis was the prevalence rate of the five included studies. The statistical analysis was performed using the statistical software R Metafor. The calculation of heterogeneity was performed by i squared (I^2^), adopting fixed effects model, considering that all the studies had the same outcome. Analyzes in which I^2^ presents value > 70% can be considered with high heterogeneity^13^ ^-^ ^14^.

There was no need to approve the study project in the Research Ethics Committee because it was a systematic review with search for scientific evidence published in databases. 

## Results

Five studies were included for the review and meta-analysis, according to Figure 2. All articles that did not present prevalence data of relapse were excluded because they were out of the scope of the research or because of the type of design.

Data were extracted from the studies selected for review, according to Figure 3. Two studies were excluded due to the use of secondary data obtained from health surveillance information systems. 

The sample of the five studies totaled 1,308 people, ranging from 70 to 586 patients with malaria diagnosed by different types of *Plasmodium*, with the majority of cases being *Plasmodium vivax.*

The prevalence of relapse was 0.47 and ranged from 17 to 92.85%, with a confidence interval of 95%^15^ ^-^ ^19^. The methodological procedures for recruitment and confirmation of relapse cases varied among the studies, and all five presented laboratory test as standard.

The meta-analysis expresses the prevalence of relapse, according to Figure 4, with a confidence interval of 95%.

The heterogeneity has I^2^ of 99.04%, with p <0.001 and 95% confidence interval (0.434, 0.509). The difference between the studies can be considered high.

## Discussion

The results show that malaria relapse is a worrisome phenomenon, since it can contribute to the disease remaining endemic in the affected areas and introduce it in non-affected areas, which can exacerbate the epidemiological picture and generate impacts on economic and social aspects^16^.

A study carried out in the city of Porto Velho, in the state of Rondônia, Brazil, indicated that the risk of relapse was estimated at 45.1/100 inhabitants and that this risk can be considered a high rate for a city of this size when compared to the rate for the Amazon region, estimated at 20.8%^8^. In Ethiopia, a study showed that 77% of secondary malaria cases are due to relapses^20^. 

It is most likely that the cause of relapse is the reactivation of the *hypnozoites* and the great capacity of *Plasmodium vivax* to adapt biologically, with phenotypic changes^7^.

Also related to the biological factor of the parasite, a study carried out in Nepal estimated the prevalence rate of relapse at 17%, stating that there may be a relationship between the specific genotypes of *Plasmodium vivax*, which may differ between the different geographical regions, considering the biological aspects of the immunity of specific populations^19^.

Knowing the prevalence of relapse is crucial for public health, since it extends the possibilities of management by malaria control programs, allowing the understanding of the magnitude of the disease and, therefore, the adequate planning of actions by services, conferring greater effectiveness to health interventions. From the epidemiological point of view, it allows users who remain with the disease, thus being sources of infection, to be identified in a timely manner, interrupting the transmission cycle^21^.

In order to verify the prevalence of relapse, it is necessary that Cure Slide Verification (CSV) be performed after the end of treatment. This is, therefore, a technology that should integrate the actions of the programs, since it is an important indicator for the health services for being able point operational problems of epidemiological surveillance, inform which users are sources of infection, “besides being useful to differentiate a new infection (new case)” from a relapse^21^.

Because it is a neglected disease, including its financing, there is an intimate relationship between its occurrence and the social and economic development of the affected region. In countries with poor populations and less access to goods and services, vulnerability to relapse is greater due to, among other reasons, limitations on financial resources for disease control programs. Thus, it can be observed that social inequality patterns determine and are determined by the occurrence of malaria^22^.

The disorderly occupation of territories by poor populations in search of subsistence, without other alternatives, or by groups with economic interests in the extraction of natural resources has been associated to the maintenance of the prevalence of neglected diseases like malaria. In this sense, the role of nurses is of utmost importance, since in some territories this is the only health professional that is present and capable of establishing control and prevention measures^4^.

The result found in the meta-analysis of the prevalence of relapse shows high heterogeneity, 99.04%. This is due to the fact that the original studies present significant differences in their methodological designs. There is still a lack of methodological standardization for these types of studies, even when appropriate techniques are applied for proper and judicious selection and eligibility. The attempt to reduce publication bias in the meta-analysis was based on the extensive search in the databases^14^.

In Brazil, the National Malaria Control Program (PNCM in Portuguese) aims to reduce the incidence and severity of malaria and, consequently, the number of hospitalizations and deaths resulting therefrom, with an increasing emphasis on studies related to the systematization of vector control processes and actions. Faced with the challenges imposed by the current times, it is necessary to “make efforts to acquire knowledge and skills to take care of new paradigms that contemplate the entirety of the individual and their insertion and inseparability with the environment^23^. 

Malaria relapse can increase disease management costs and affect the effectiveness of disease control programs. Considering its relation with poverty and underdevelopment, one can question how the poorest countries control new cases and relapse, since some studies indicate that economic and social development is directly associated with the prevalence of neglected diseases^24^.

Malaria relapse is an important indicator of the outcome of the control programs, since it can increase disease management costs and affect the effectiveness of their actions. Another important aspect is that the relapse rate points to the need to monitor the resistance of the parasite to antimalarials and can help to promote changes in drug policies.

A more specific look at relapse cases is also necessary due to its relevance for the maintenance of the endemic disease, its high prevalence rate and consequent challenges for health surveillance^25^.

The study presents limitations because the gray literature was not consulted and because of its high heterogeneity.

The results of this work can be used as a basis for conducting research on economic evaluation of health technologies to intervene in malaria.

## Conclusion

Malaria, even with advances in technology and epidemiological science, remains a challenge for public health. The phenomenon of malaria relapse has contributed to the fact that its endemicity has not changed and compromises the effectiveness of programs aimed at its control or even eradication. 

The prevalence of relapse is concerning not only from the clinical point of view, in which the damage to the patient is directly expressed in their non-cure, their lack of conditions to work and consequently in the prejudice of their quality of life. 

In addition to this factor, there are also the slow and insufficient progress in the development of therapeutic alternatives and other technologies that are capable of braking the transmission of the disease, the financial investments also insufficient for the new cases and for relapse cases, human action on the environment and the biological capacity of the etiological agent to adapt and produce genetic changes that challenge the combat of the disease.

The estimated prevalence in this study points to the need to establish measures that go beyond the field of care, including the area of professional training, and scientific research and development. Outlining policies and programs to control neglected diseases, such as malaria, require the understanding of the magnitude of phenomena such as relapse so that they can be truly effective, meeting the needs of the population. 
